# The ovarian germinal reserve and apoptosis-related proteins in the infant and adolescent human ovary

**DOI:** 10.1186/s13048-019-0496-2

**Published:** 2019-03-11

**Authors:** María Itatí Albamonte, Mirta Susana Albamonte, Ricardo M. Bou-Khair, Luis Zuccardi, Alfredo Daniel Vitullo

**Affiliations:** 1 Centro de Estudios Biomédicos, Biotecnológicos, Ambientales y Diagnóstico –CEBBAD Universidad Maimónides, C1405BCK Buenos Aires, Argentina; 2 Consejo Nacional de Investigaciones Científicas y Técnicas, CONICET, C1425FQB Buenos Aires, Argentina; 3 Servicio de Ginecología Infantil, Hospital de Niños “Dr. Ricardo Gutiérrez”, C1425EFD Buenos Aires, Argentina

**Keywords:** Human ovary, Adolescence, Ovarian reserve, Apoptosis, BCL2-family proteins, OCT3/4, VASA, FAS-FAS-L

## Abstract

**Background:**

Normal pubertal ovary displays all stages of follicular development and a biased BAX/BCL2 protein ratio in favor of pro-apoptotic BAX protein comparable to the adult ovary. However, adolescents suffering malignant extra-gonadal disease show a limited follicle development after cytotoxic drug treatment and a reduced capacity of in vitro follicle growth. We evaluated the expression of pro- and anti-apoptotic members of the *BCL2* gene family, the FAS/FAS-L proteins from the extrinsic apoptosis pathway, the germ-cell-specific marker VASA, the pluripotency marker OCT3/4, and markers of early and late apoptosis in the ovary of pubertal patients with malignant extra-gonadal disease, which received or not pre-surgery chemotherapy, entering a cryopreservation program.

**Results:**

Ovarian biopsies from 12 adolescent girls were screened for follicle count and expression of VASA, OCT3/4, BAX, BCL2, MCL1L and S, cleaved-BID, FAS/FAS-L and CASPASE 3 through immunohistochemistry, western blot and RT-PCR. All stages of folliculogenesis, from primordial to antral follicle, were present in all 12 patients analyzed. VASA and most of the screened apoptosis-related genes showed a pattern of immune-expression comparable to that previously reported. OCT3/4 showed a cytoplasmic localization in the great majority of the primordial follicles; however, in some cases the localization was nuclear. In addition, OCT3/4B showed a significant reduction compared to OCT3/4A. Unexpectedly, BCL2 was detected at all stages of folliculogenesis, associated to the Balbiani’s body in the primordial follicles, regardless of whether patients had or had not received chemotherapy, ruling out the possibility that its expression is a protective response to chemotherapy.

**Conclusions:**

These findings reveal new information on the morphological status of the follicular reserve and the expression of apoptosis-related genes in histologically normal adolescent ovary from patients undergoing extragonadal cancer. The unexpected expression of apoptosis-inhibiting BCL2 protein, both in patients that had or had not received chemotherapy, opens a new avenue for thorough investigations. Moreover, the nuclear localization of OCT3/4 protein in primordial follicle-enclosed oocytes suggests a possible increased activity of ovarian stem cells in response to chemotherapy and/or extragonadal cancer. This new information can be essential for a better managing of in vitro culture of follicles that can be removed by filtration from preserved ovarian tissue, especially in girls that entered a cryopreservation program.

## Background

In women, the establishment of the germinal reserve takes place during fetal life through the interplay between germ cell proliferation and death in the developing ovary. As soon as proliferation begins to increase the 1000–2000 primordial germ cells that reached the developing ovary, to produce around 7 × 10^6^ potential oocytes at mid-fetal life, a counterbalance mechanism of cell death, executed mainly through apoptosis, takes place. The size of the resting primordial follicle reserve is determined by the fourth month of fetal life, as a consequence of cell proliferation/cell death balance, when primordial follicle development begins. Germ cell attrition particularly occurs in the transition from meiotic prophase I to primordial follicle formation through massive apoptosis and continues until the end of gestation, leading the developing ovary to contain just around 1-2 × 10^6^ primordial follicles at birth; a wasteful cellular loss of almost 85% of the potential 7 × 10^6^ oocyte population reached at mid gestation [8, 16]. Death is mainly driven through intrinsic apoptosis mechanisms governed by a concerted expression of *BCL2* gene family members [1] acting in the germ cell proper throughout fetal life [4, 36]. In the adult ovary, germ cell elimination through apoptosis will continue, both in resting and growing follicles, acting mainly in granulosa cells surrounding the oocyte, until the germinal reserve is exhausted and women enter menopause [3]. Although apoptosis occurs under physiological conditions and contributes to maintain cellular homeostasis, an alteration in its regulation may lead to tissue alterations. For example, it was observed that a decrease in apoptosis is present in endometriosis and ovarian cancer [7, 14, 37].

In the last 20 years, several studies analyzing the expression of *BCL2*-family genes have focused mainly in the fetal and adult ovary, showing that pro-apoptotic members prevail throughout ovarian development, determining the known high rates of oocyte elimination whereas anti-apoptotic genes, like *BCL2* and *MCL1*, show a time-restricted pattern of expression and act as key regulators determining survival and preserving germ cell availability at proliferative time-points [18, 35, 36]. The infant and adolescent ovary deserved less attention probably due to the limited availability of samples. Histological studies performed in the 70s’ have shown that all stages from primordial to early-antral follicles are detected in the infant ovary [27, 28], with the exception of the pre-ovulatory follicle that appears at the onset of puberty [29], showing that follicle growth is by and large comparable to the adult ovary. However, follicle development is inhibited in children with abdominal tumors or treated with cytotoxic drugs or irradiation [20, 21]. More recently, an analysis of children and adolescent ovary from patients with malignant or chronic illness showed a high proportion of abnormal non-growing follicles with reduced capacity for in vitro development [6]. We have previously analyzed BCL2 and BAX proteins in normal infant and pubertal ovary showing that both proteins behave as in fetal life as far as the oocyte remains in the primordial resting reserve, whereas in follicles entering the growing pool gene expression moves from the germ cell to granulosa cells with an expression pattern comparable to the adult ovary [2]. Nevertheless, no data exist so far regarding the behavior of apoptosis-related proteins in the adolescent ovary from patients suffering malignant extra-gonadal disease whose ovary has been shown to have limited follicle development after cytotoxic drug treatment or irradiation [20, 21] and display a reduced capacity of in vitro follicle development [6].

The aim of this study was to evaluate the expression of pro- and anti-apoptotic members of the *BCL2* gene family, the FAS/FAS-L proteins from the extrinsic apoptosis pathway, the germ-cell-specific marker VASA, the pluripotency marker OCT3/4, and markers of early- and late-apoptosis in the ovary of pubertal patients with malignant extra-gonadal disease, which received or not pre-surgery chemotherapy, entering a cryopreservation program.

## Results

### Follicle count in cortical ovarian fragments

Active folliculogenesis was found in all samples analyzed. The ovary from the 7 years old patient (pre-pubertal, sample 1) showed primordial, primary, secondary, antral and atretic antral follicles (Table 1). The ovaries from 12 to 19 years old pubertal patients showed all follicular stages from primordial follicle to antral follicles, atretic antral follicles and luteum and albicans bodies (Table 1). Although tissue volume and the total follicle count showed a high variability among patients, primordial follicle was the more abundant follicular stage representing > 50% of the total count, reaching more than 80% in most cases (Table 1). Mean percent number of primordial follicles did not show statistical differences between patients that had (90.25 ± 10.53%) or had not (82.63 ± 17.72%) received chemotherapy.

**Table 1:** Follicle count and follicle classification in adolescent human ovarian biopsies from patients with extra-gonadal malignant disease

Patient	Number of follicles					Tissue volume (mm^3^)	Follicular pool	Primordial follicles (%)
	Primordial	Primary	Secondary	Antral	Others			
1	343	30	4	1	1 atretic	100	379	90,5
2	1060	68	7	1	2 luteum bodies	23.4	1138	93.14
3	56	47	2	0	2 atretics; 2 luteum bodies; 1 albicans body	84	110	50,9
4	533	4	1	0	4 luteum bodies	80	542	98.3
5	1295	22	6	0	2 atretics, 1 luteum body, 2 albicans bodies	52.5	1328	97.52
6	3555	154	7	1	1 atretic; 1 luteum body; 2 albicans bodies	101,5	3721	95,5
7	326	50	5	3	1 atretic; 2 luteum bodies; 3 albicans body	192	390	83,6
8	4081	73	17	0	1 atretic; 1 luteum body; 1 albicans body	108	4174	97,8
9	2178	1	1	0	1 albicans body	360	2181	99.9
10	72	13	3	0	1 luteum body	94.5	89	80.9
11	111	26	6	0	3 atretics y 1 luteum body	168	147	75.5
12	233	59	20	1	1 atretic y 2 luteum bodies	21	316	73.73

### Expression of VASA and OCT3/4 proteins

**Fig. 1:**
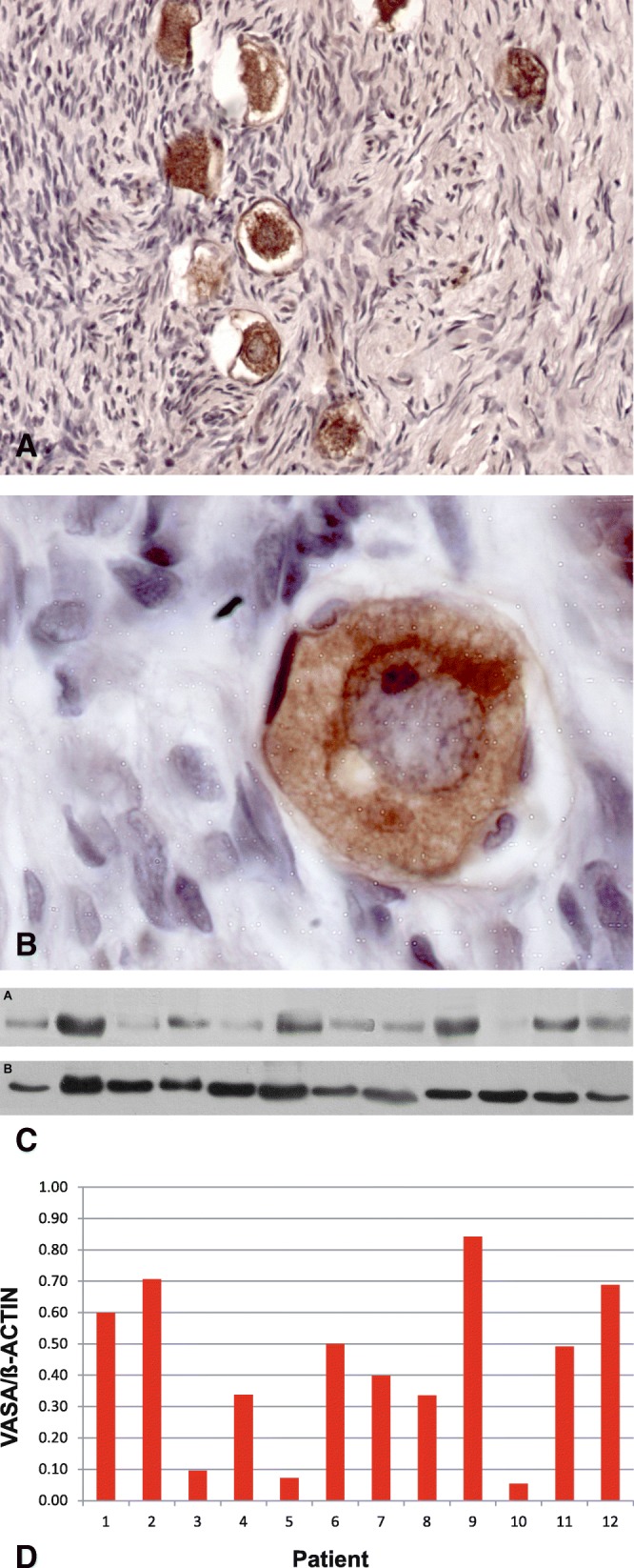
Detection of germ-cell-specific VASA protein in human ovary. **a** Primordial follicles VASA positive in cytoplasm oocyte (200X). **b** Primordial follicle VASA positive with para-nuclear localization corresponding to Balbiani’s vitelline body (1000X). **c**) Western blot analysis of human ovarian VASA protein (79 kD). **d** Western blot quantification of VASA protein. This protein was detectable in all samples with variable intensity

**Fig. 2:**
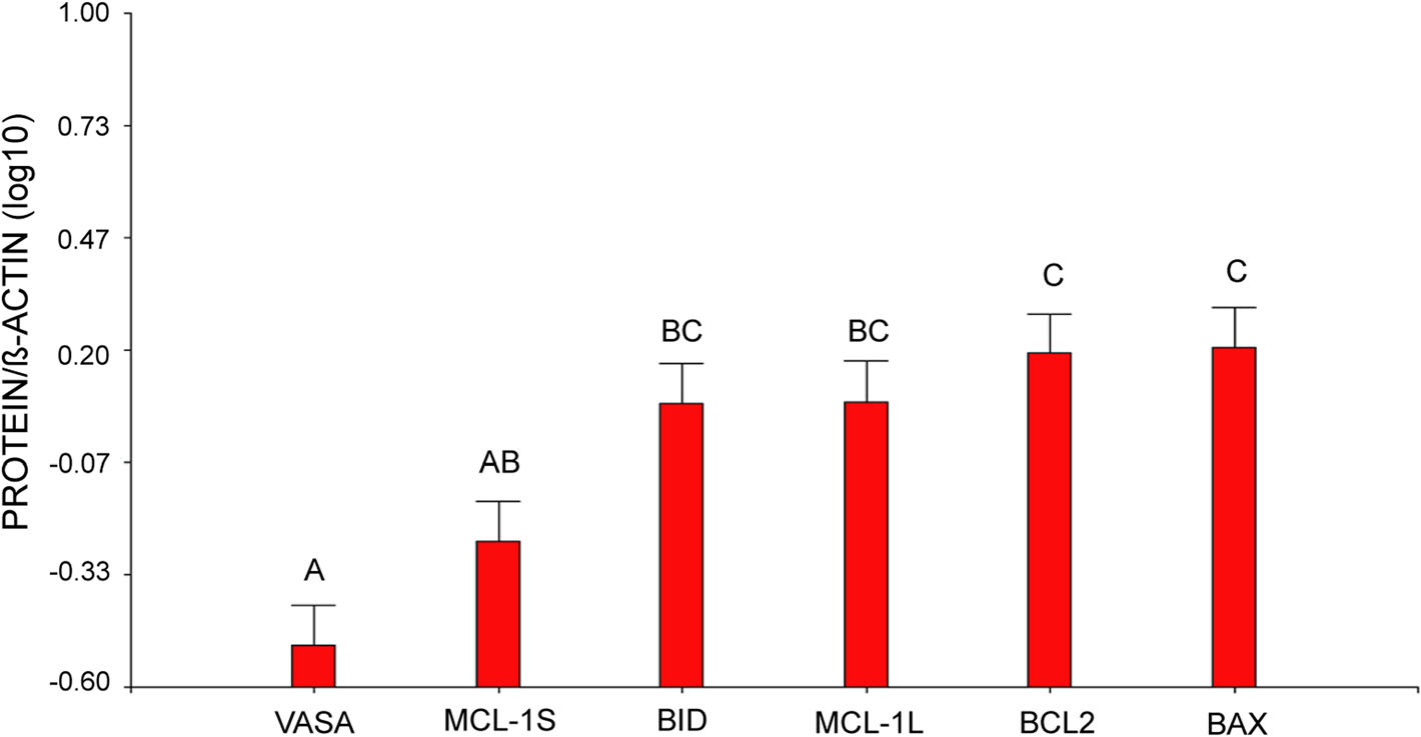
Western blot statistical analysis for VASA, BAX, BCL2, MCL-1 and cleaved-BID proteins in human ovary from pre- and pubertal oncological patients. Each bar represents a mean ± s.d. Different letters over the bars indicate significant differences between samples (Tukey, α: 0,05, p < α)

Germ-cell-specific VASA protein was detectable in the cytoplasm of oocyte in primordial and primary follicles (Fig. 1a); no signal for VASA was found in somatic stratum. Advanced primordial follicles showed VASA protein in a para-nuclear localization corresponding to Balbiani’s vitelline body (Fig. 1b). In primary follicles, VASA distributed throughout the oocyte cytoplasm. VASA immune detection was corroborated through protein analysis (Fig. 1c) in variable amounts among patients (Fig. 1d) and its expression was significantly diminished compared to BID, MCL-1 L, BCL2 and BAX proteins (Tukey test, α:0.05; *p* < 0.0001) (Fig. 2).

**Fig. 3:**
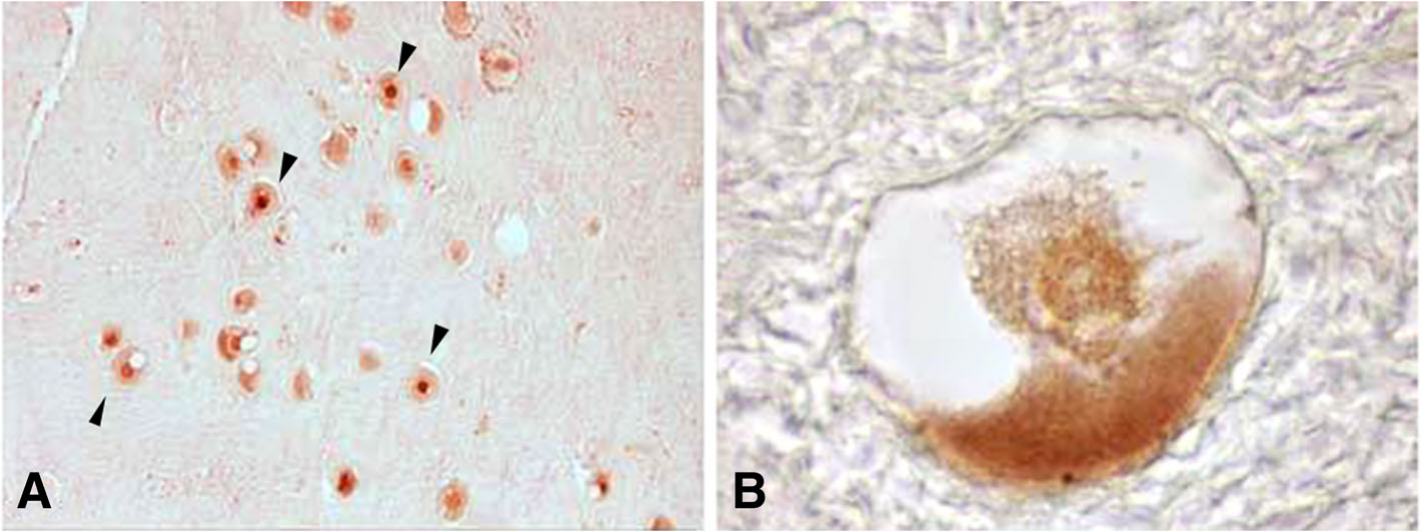
Immune-localization of OCT3/4 protein in adolescent human ovarian tissue. **a** Primordial follicles OCT3/4 positive. Note some follicles were positive in oocyte cytoplasm and others were positive in nucleus (Arrows head) (100X). **b** Detailed view of a typical primordial follicle; OCT3/4 protein was detectable in the cytoplasm of oocyte (1000X)

OCT3/4 protein was mainly detected in the cytoplasm of oocyte in primordial, primary and secondary follicles (Fig. 3). However, nuclear signals in occasional primordial follicle-enclosed oocytes were also detected in patients 1, 6 and 9 (Fig. 3a).

### Expression of intrinsic apoptosis proteins

**Fig. 4:**
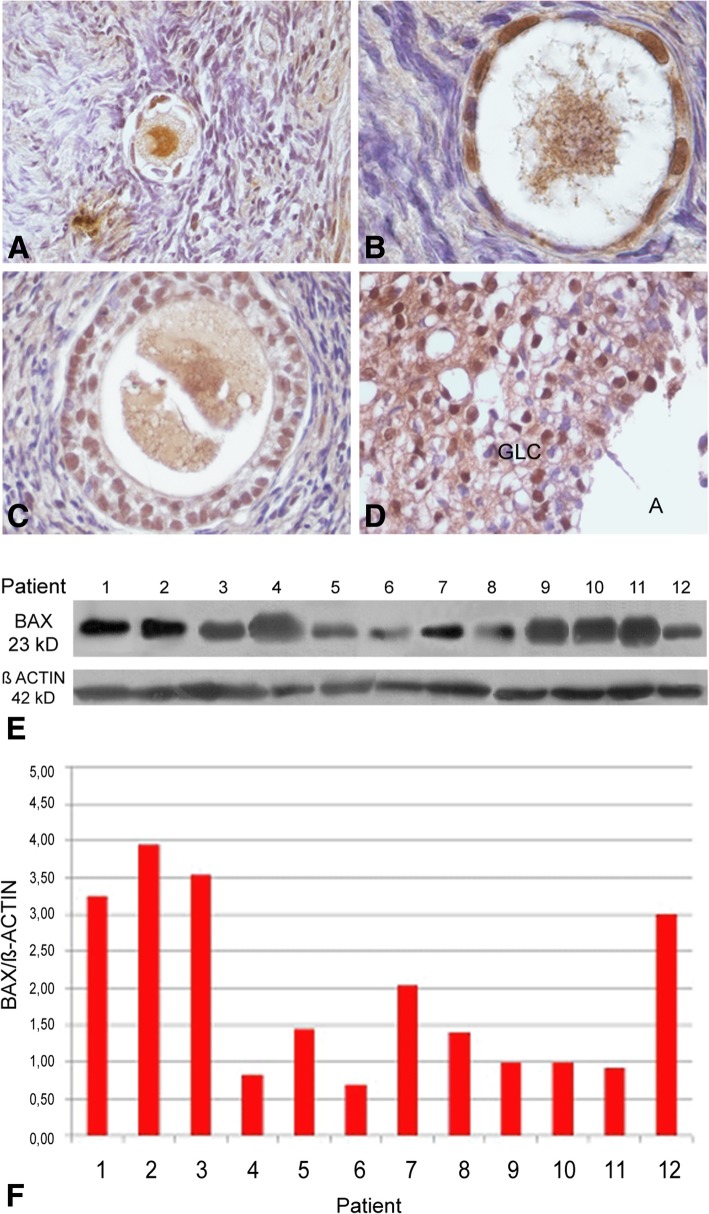
Detection of pro-apoptotic BAX protein in adolescent human ovary. **a** Primordial follicles show BAX protein in granulosa cells and in the germ-cell proper (200X). **b** The signal persists in primary follicle (1000X) and **c**) secondary follicle (400X). **d** Partial view of a luteum body positive for BAX (400X). **e** Western blot analysis of human ovarian BAX protein (23 kD). **f** Western blot quantification of BAX protein. This protein was detectable in all samples. GLC: granulosa-luteal cells

**Fig. 5:**
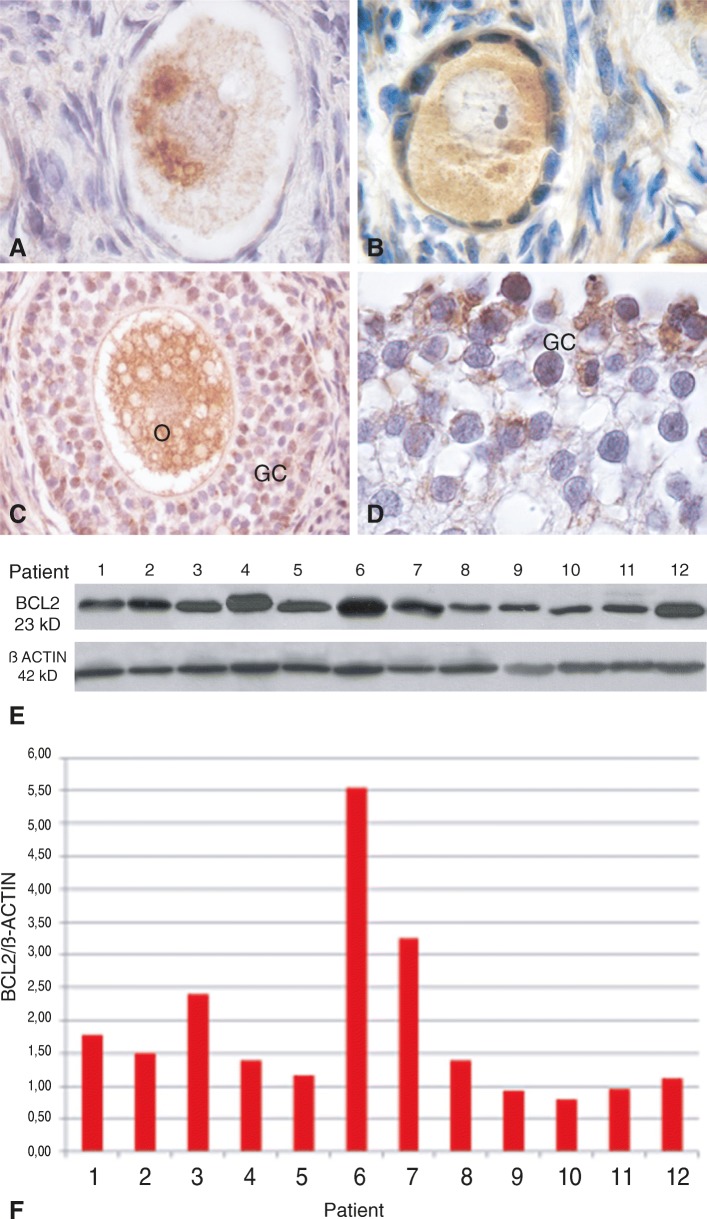
Detection of anti-apoptotic BCL2 protein in adolescent human ovary. **a** Primordial follicle and **b**) primordial/primary follicle BCL2 positive. BCL2 was heterogeneously distributed in a para-nuclear localization in the oocyte cytoplasm (Arrow heads) (1000X). **c** Secondary follicles show BCL2 protein in granulosa cells and in the germ-cell proper (200X). **d** Antral follicle (partial view), BCL2 is detectable in granulosa cells (1000X). **e** Western blot analysis of human ovarian BCL2 protein (23 kD). **f** Western blot quantification of BCL2 protein. It was detectable in all samples with variable intensity. O: oocyte; GC: granulosa cells

Immunolocalization of intrinsic apoptosis proteins was restricted to the follicular compartment without detection in the interstitial environment. The pro-apoptotic BAX protein was detectable in all follicular stages. BAX signal was detected both in the cytoplasm of the oocyte as in granulosa cells from early primordial to secondary follicle (Fig. 4a-c); in antral follicles, BAX detection was mainly restricted to granulosa. Corpora lutea and albicans bodies also displayed BAX signal (Fig. 4d). BAX protein immunoblot was positive in all patients with variable levels among them (Fig. 4e, f). Immunolabelling with anti-BCL2 was restricted to the oocyte in early primordial follicles (Fig. 5a); it was detectable in the oocyte and granulosa cells of primordial/primary follicles (Fig. 5b) and mostly detected in granulosa cells in more advanced stages (Fig. 5c, d). BCL2 distributed heterogeneously in the oocyte cytoplasm, in a para-nuclear localization, in primordial and primordial/primary follicles (Fig. 5a, b). BCL2 protein immunoblot was positive in all patients, although highly variable among them (Fig. 5e, f). Statistical analysis did not show significant differences between BAX and BCL2 proteins (Fig. 2).

**Fig. 6:**
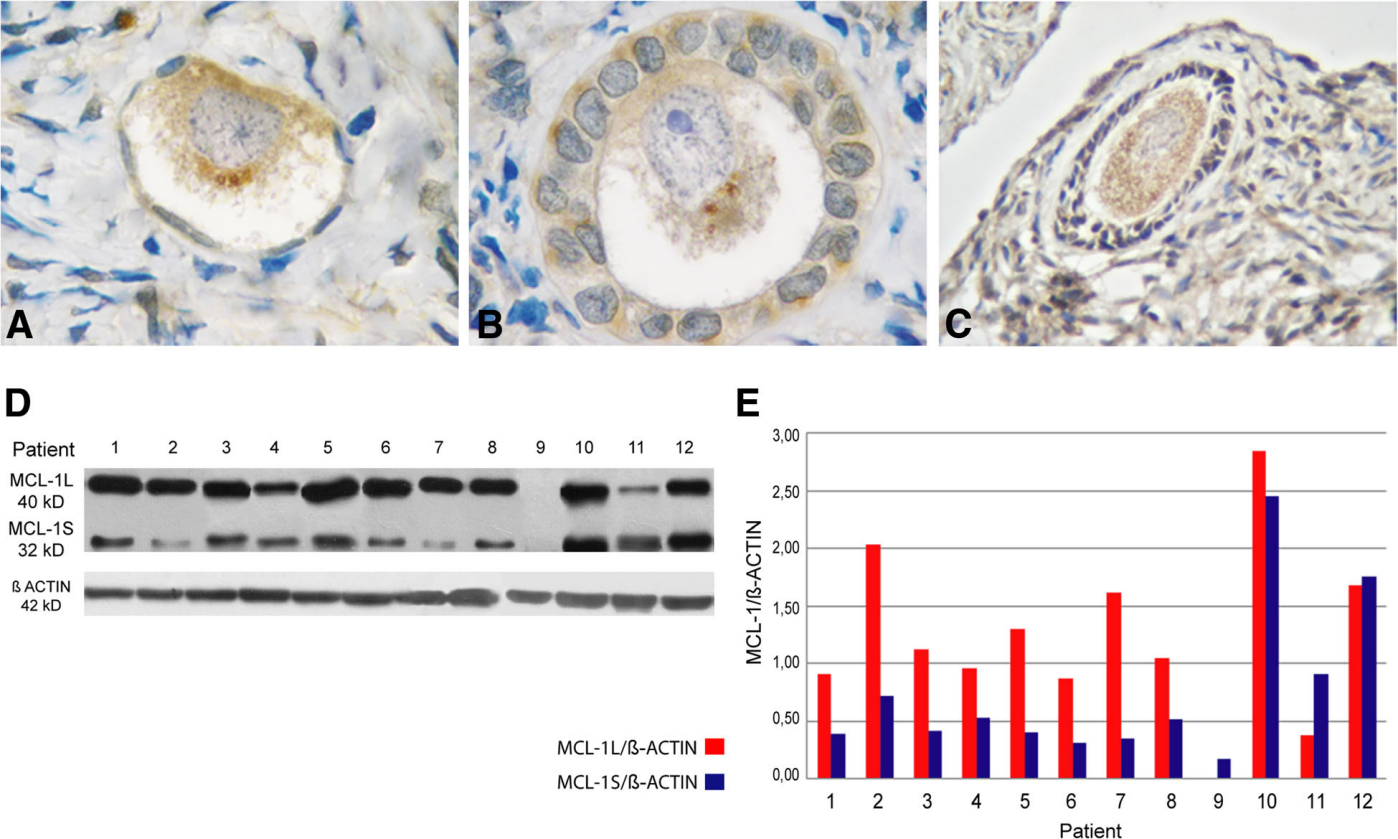
Detection of MCL-1 protein in adolescent human ovary. **a** Primordial follicle (1000X), **b** primary follicle (1000X) and **c**) secondary follicle (200X) show MCL-1 protein in granulosa cells and in the germ-cell proper. **d** Western blot analysis for both isoforms of MCL-1, the anti-apoptotic MCL-1 L (40 kD) and the pro-apoptotic MCL-1S (32 kD). **e** Western blot quantification of MCL-1 L and MCL-1S proteins. Both isoforms were detectable in all samples with variable intensity

MCL-1 protein was detected in the cytoplasm of oocytes in early primordial follicles with a heterogeneous distribution (Fig. 6a). In more advanced stages, e.g., primary, secondary and preantral follicles, the signal remained in the oocyte cytoplasm and it was also found in granulosa cells (Fig. 6b, c). Western blot analysis distinguished between anti-apoptotic MCL-1 L and pro-apoptotic MCL-1S variants (Fig. 6d). Both MCL-1 isoforms were detected in all cases, with the exception of patient 9. In the majority of cases (patients 1–8 and 10) the anti-apoptotic isoform prevailed and only in patients 11 and 12 the pro-apoptotic variant was favored in the face of the anti-apoptotic isoform (Fig. 6e). The statistical analysis for both isoforms did not show significant differences between them (Fig. 2).

**Fig. 7:**
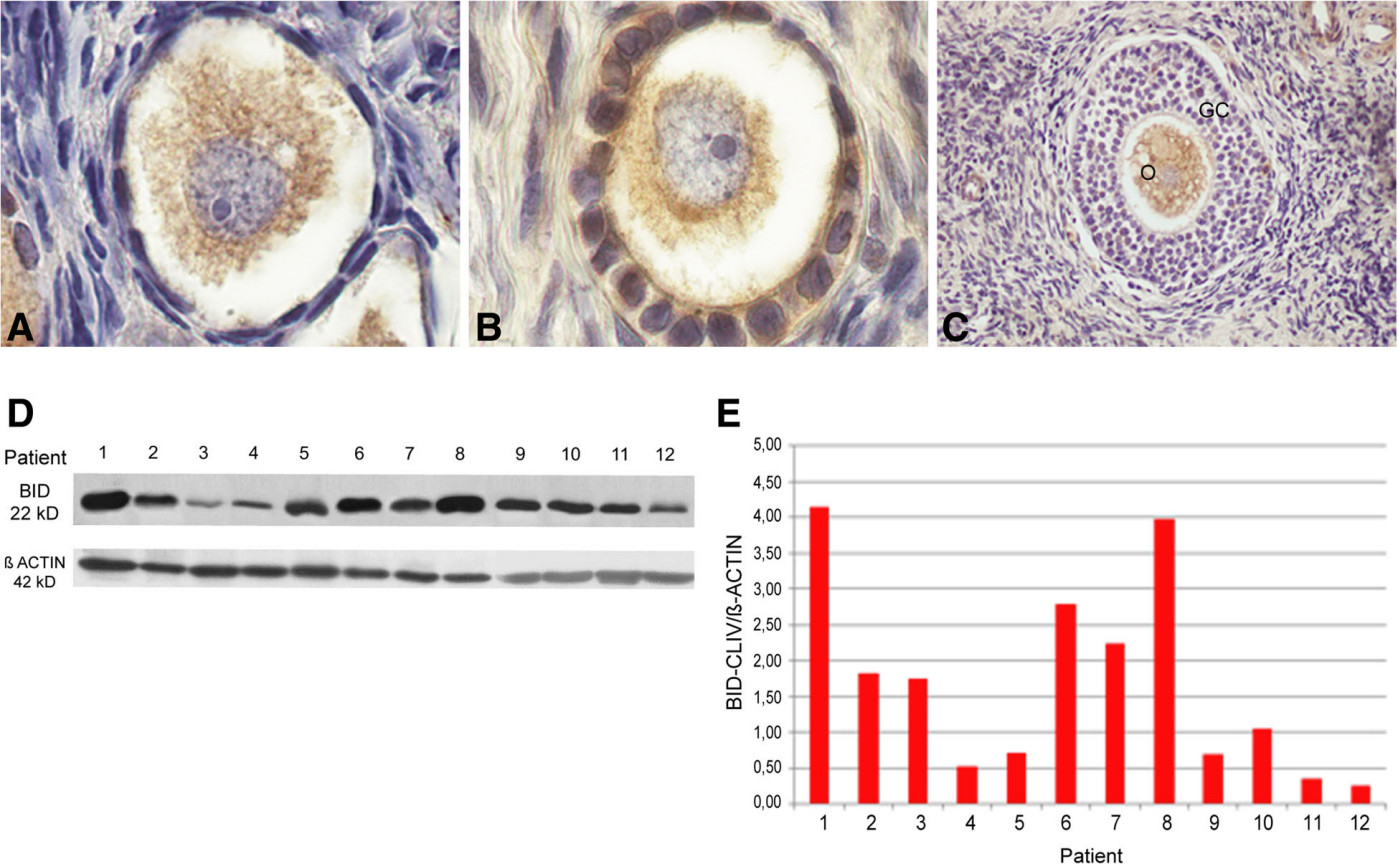
Detection of pro-apoptotic cleaved-BID protein in adolescent human ovary. **a** Primordial follicle and **b**) primary follicle cleaved-BID positive (1000X). **c** Secondary follicles show cleaved-BID protein in granulosa cells and in the germ-cell proper (200X). **d** Western blot analysis of human ovarian cleaved-BID protein (22 kD). It was detectable in all samples. **e** Western blot quantification of cleaved-BID protein. It was detectable in all samples with variable intensity. O: oocyte; GC: granulosa cells

The pro-apoptotic cleaved-BID protein was detected mainly in granulosa cells and oocytes in primordial and primary follicles (Fig. 7a, b) and the signal still persisted in the oocyte cytoplasm of some secondary follicles (Fig. 6c). Cleaved-BID protein immunoblot by western blot was positive in all cases with variable amount among patients (Fig. 7d, e). No differences in cleaved-BID recovery was found when compared with other proteins, except with VASA (Tukey test, α: 0.05; *p* < 0.0001) (Fig. 2).

### Immunolocalization of extrinsic FAS/FAS-L proteins

**Fig. 8:**
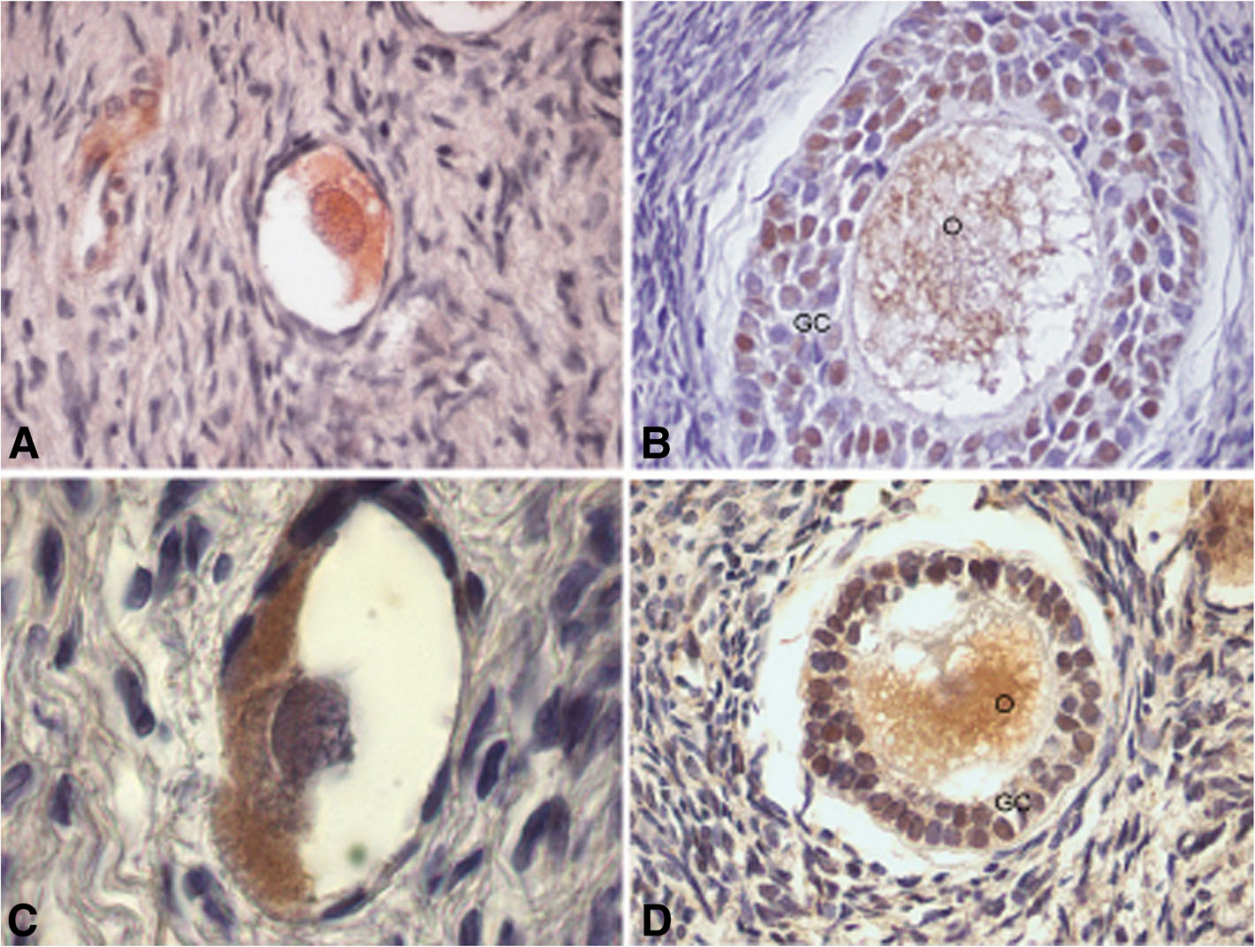
Immunostaining for both FAS/FAS-L proteins in adolescent human ovary. **a** Primary follicle (400X) and **b**) secondary follicle (400X) were positive for FAS protein. In the last one, it was detectable in oocyte and granulosa cells. **c** Primordial follicle positive for FAS-L in cytoplasm oocyte (1000X). **d** Secondary follicle positive for FAS-L in oocyte and granulose cells (400X). O: oocyte; GC: granulosa cells

FAS/FAS-L proteins were positive in primordial, primary and secondary follicles both in oocyte and granulosa cells (Fig. 8a-d) and they were detected in antral follicles, corpus luteum and the albicans body as well.

### Caspase-3 detection and TUNEL assay

**Fig. 9:**
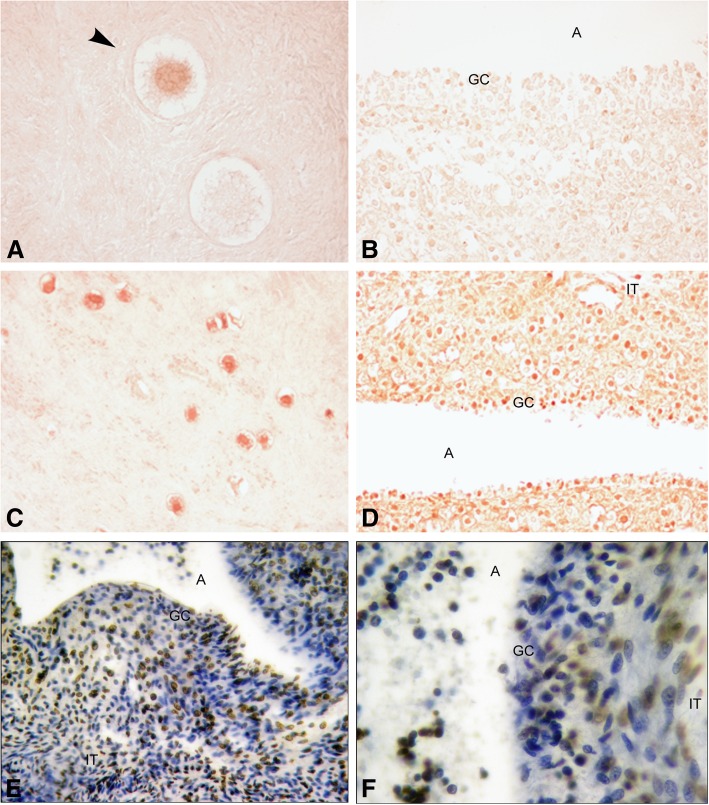
Immunodetection for CASPASA-3 and TUNEL-assayed in human ovary. **a** Primordial follicles positive (arrow head) and negative for pro-caspase 3 (400X). **b** Partial view of an atretic antral follicle negative for pro-caspase 3 (200X). **c** Panoramic view of cortex ovary showing abundance of primordial follicles positive for cleaved caspase 3 protein (100X). **d** Atretic antral follicle was positive for cleaved caspase 3 in granulosa and thecal cells (200X). **e** Apoptotic cells were detected in granulosa and thecal cells of antral follicles (400X). **f** Partial view of an atretic antral follicle with granulosa cells TUNEL positive (1000X). A: antrum; GC: granulosa cells, IT: internal theca

Pro-caspase 3 was positive in some primordial follicles (Fig. 9a). The protein was also detected in some primary and secondary follicles. Atretic antral follicles were negative (Fig. 9b). The majority of primordial follicles were positive for cleaved caspase-3 (Fig. 9c) which was also detected in primary, secondary and atretic antral follicles (Fig. 9d). Apoptotic cells were detected by TUNEL in the innermost layer of granulosa cells in antral follicles, mainly in late antral stage. A few apoptotic theca cells were positive in antral follicles (Fig. 9e, f).

### mRNA expression in adolescent ovary

**Fig. 10:**
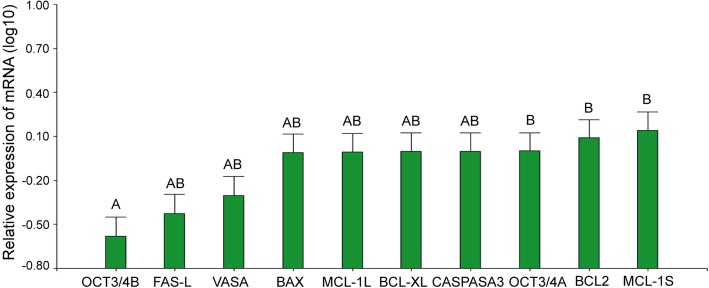
mRNA expression in human ovary from pre- and pubertal oncological patients. Each bar represents a mean ± s.d. Different letters over the bars indicate significant differences between samples (Tukey, α: 0,05, p < α)

The expression of *VASA*, *OCT3/4 A* and *B*, *BAX*, *BCL2*, *MCL-1 L* and *S*, *BID*, *BCL-XL*, *FAS*, *FAS-L* and *CASPASE 3* were similar among the analyzed patients. Most of the genes did not show significant differences, except *OCT3/4B* whose expression was significantly diminished with respect to *OCT3/4A*, *BCL2* and *MCL-1S* expression (Tukey, α:0.05; *p* < 0.0001) (Fig. 10b).

## Discussion

Our current knowledge about the infant and teen human ovary is still scarce, most likely due to difficulties in obtaining samples from children and adolescents. Besides reports on the general histology performed in the 70s’ [20, 21, 27–29], a few studies have been published more recently analyzing the ovarian histogenesis, follicular composition and immunolocalization of apoptosis- and germline-related proteins [2, 6, 15].

### The ovarian reserve and folliculogenesis in the pubertal ovary

Ovarian sections from all 12 patients examined showed an active folliculogenesis and a high density of primordial follicles. The wide variability of primordial follicles, ranging from 50 to 99% of the total follicular pool, among patients (cf. Table 1) may be attributed to the random and the heterogeneous distribution of follicles in the human ovary [16, 32], especially considering that primordial follicles are normally found in clusters [31]. In agreement with previous studies [15], pubertal ovaries showed all follicular stages, including antral follicles and luteum and albicans bodies as well, confirming the post-menarche beginning of cyclic follicle recruitment. The only pre-pubertal ovary included in this report, a 7 years old patient, showed that histogenesis was still in progress with initial follicle recruitment. We did not observe significant differences in the percentage of primordial follicles between patients that had or had not received chemotherapy before surgery. Although chemotherapy drugs are known to be highly toxic and they can upregulate the PI3K pathway leading to a wave of follicular recruitment and growth that burnout the ovarian reserve [23], our results agreed with Duncan et al. [15] in that the adolescent ovary displays follicles irrespective of treatment history.

### VASA, OCT3/4 and apoptosis-related proteins in the adolescent ovary

VASA expression was restricted to the cytoplasm of oocytes in primordial and primary follicles in all 12 patients analyzed. In primordial follicles, VASA showed a para-nuclear localization corresponding to Balbiani’s vitelline body, whereas in primary follicles VASA distributed homogeneously in the cytoplasm. No difference with previous observations was detected [2]. It has been proposed that VASA expression follows a stage-specific immune detection pattern that reflects the main changes during fetal ovarian development [4, 5, 12, 34]. In post-natal ovaries, we have found that VASA continues to express after birth until puberty in the resting primordial follicle reserve, associated with the Balbiani’s body; its expression pattern relates mainly to the follicular stage rather than to the stage of development [2]. The association or not of VASA to the Balbiani space may relate to the final fate of the germ cell [4]. In support, the ovary of *Lagostomus maximus*, a rodent with no germ cell attrition or apoptosis-dependent follicular atresia through constitutive expression of *BCL2*, shows a low abundance of Balbiani-associated VASA throughout development [26]. The presence of anti-apoptotic BCL2 in Balbiani’s body (see below) in patients with extragonadal cancer might influence the survival of the primordial follicle.

**Fig. 11:**
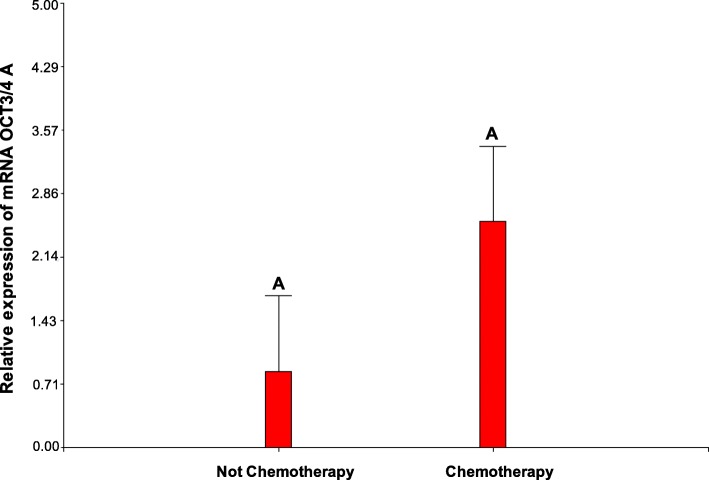
OCT3/4-A gene activity in patients that had or had not received chemotherapy. No significant statistical differences were detected between groups; however, gene activity tends to be increased in response to chemotherapy

OCT3/4 protein was detected from primordial to secondary follicles in the adolescent ovary with an expression of isoform A significantly increased in the face of isoform B (cf. Fig. 10b). Moreover, *OCT3/4-A* gene activity showed a tendency to be increased in patients that received chemotherapy compared to those that did not (Fig. 11). Although no statistical differences were detected among these groups, the small size of samples must be considered. OCT3/4 is a POU domain transcription factor involved in the regulation of cell pluripotency and renewal [25]. The expression of OCT3/4 isoforms has been related to two different populations of stem cells residing in the ovarian surface epithelium: *very small embryonic-like stem cells* (VSELs), small-sized pluripotent cells that express nuclear OCT3/4-A and give rise to *ovarian stem cells* (OSCs), slightly bigger than VSELs, expressing cytoplasmic OCT3/4-B [9, 10]. The detection of nuclear OCT3/4 in three patients in this study together with the increased expression of isoform A versus isoform B suggest an increased activity of stem cells in these ovaries. If the presence of nuclear OCT3/4 is a response to chemotherapy, to the presence of extra-gonadal cancer or both requires further analysis. Only two of the three patients who presented nuclear OCT3/4 had received chemotherapy. Both chemotherapy and the presence of cancer in any organ of the body compromise homeostasis and may be sufficient to trigger the activity of stem cells [11].

Our analysis of BCL2 family proteins showed that the immunolocalization pattern of pro- and anti-apoptotic members was related to follicular stage. BAX and MCL-1 were detectable throughout folliculogenesis whereas cleaved-BID was restricted to the follicular reserve. The pattern of immunolocalization of BAX was similar to that we previously observed in infant, pubertal and adult ovaries [2, 3]. Unexpectedly, the anti-apoptotic BCL2 protein showed an immunolocalization quite different to that previously reported. This protein was detectable throughout folliculogenesis, including primordial and primary follicles of the germinal reserve. The expression of BCL2 in the primordial reserve both in patients that had or had not received chemotherapy rules out the possibility that BCL2 expression may be linked to a chemo-treatment response, supporting the idea that it is related to the presence of an extragonadal tumor. In previous studies, BCL2 protein was not detectable in dormant primordial follicles and primary follicles, leaving the resting reserve in postnatal ovary from individuals not undergoing a cancer situation. It became detectable in the somatic stratum from secondary follicles, as well as in preantral and antral follicles, co-existing with BAX protein [2, 3]. It is worth to note that BCL2 becomes detectable when proliferative processes occur, i.e. at the moment in which the number of oogonia is actively increasing through mitosis in the fetal ovary or when granulosa cells actively divide during the growing follicular phase in the infant/pubertal ovary [2, 4, 18, 35, 36]. In the light of the expression pattern of OCT3/4 found in these patients, especially the presence of nuclear expression (see above), the unexpected detection of BCL2 in the germinal reserve could be a response to an increased proliferative activity of ovarian stem cells. Alternatively, BCL2 expression could be due to a protective response to the presence of an extragonadal pathological process. Cancer is, per se, a disease that induces oxidative stress, thus generating toxic effects on normal cells, combined with anoxia and deficits in nutrients and antioxidants that cannot compensate the production of free radicals [19, 33]. It is tempting to think that germ cells are responding to survive in the stressing environment. BCL2 detection could be of good prognosis for an eventual future recovery of the gametogenic capacity, considering the protective concurrent expression of OCT3/4.

The FAS/FAS-L system involved in the extrinsic pathway of apoptosis, displayed an immunohistochemical pattern comparable to that found for the apoptosis-inducing BAX protein. This system has deserved little attention, and a few contributions explored its role in the human ovary [3, 13, 24, 30]. It is worth to note that both FAS protein and its ligand, FAS-L, displayed strong immune labeling in the somatic stratum of antral and atretic follicles and the corpus luteum. These observations reinforce the idea that FAS/FAS-L acts mainly in follicular regression and atresia [24].

The occasional presence of cleaved-caspase 3 in primordial follicles suggests that the process of cell damage would be already at play in some follicles that are going to be recruited to the growing pool and most likely they will enter atresia during or before reaching the antral stage. Only antral follicles, and especially fully-grown follicles, as well as atretic follicles, showed TUNEL and cleaved-caspase 3 positive signals in granulosa and theca cells, indicating an active apoptotic process accompanying follicular atresia.

### Final considerations

A long-held tenet supports the idea that mammals’ female are born with a finite and non-renewable endowment of oocytes. Nevertheless, the detection in 2004 by Tilly’s group [22] of a small cell population in the adult mouse ovary residing in the ovarian surface epithelium and expressing meiotic markers fueled the possibility of female neo-oogenesis after birth. Fourteen years later, although some controversies still persist, the presence of ovarian stem cells that could act to regenerate the germline is a proven fact. The detection, in this report, of anti-apoptotic markers and nuclear OCT3/4 expression in the primordial reserve of adolescent patients undergoing extragonadal cancer adds new information on stem cell activity in response to stress conditions and illuminates an encouraging perspective for the restoration of fertility through the development of reliable culture techniques for in vitro re-establishment of the gametogenic capacity in girls that entered a cryopreservation program.

## Methods

### Recovery of ovarian samples

A total of 12 patients entering an ovary cryopreservation program at Hospital de Niños “Ricardo Gutiérrez”, Buenos Aires city, were included in this study. All patients had suffered extra-gonadal malignant disease and 6 of them received chemotherapy before surgery. In all these 6 patients, samples were taken one month after the end of treatment. At the time of cryopreservation, the patients were aged between 7 and 19 years and one of them (aged 7 years old) was pre-pubertal (Table 2). At the surgery room, the entire ovarian cortex was excised for cryopreservation and 3 small fragments (1.0 × 0.5 mm) were recovered for research purposes. One fragment was fixed in 10% buffered formalin; a second fragment was stored at − 80 °C and a third one was immersed in 1 ml of RNA later. All patients had a normal ovary histological diagnosis from the Pathology Service of the Hospital Ricardo Gutiérrez. The protocol for ovarian cryopreservation and donation of samples for research was reviewed and approved by the Research Ethics Committee of Universidad Maimónides, Buenos Aires, Argentina, and the Ethics Committee from Hospital de Niños “Ricardo Gutiérrez”, Buenos Aires, Argentina. Samples were used for research purposes after obtaining the patients and/or parents’ informed consent.

**Table 2:** Diagnosis, age and pre-surgery treatment in oncological patients included in the present study

PATIENT	AGE (years)	DIAGNOSIS	PRE-SURGERY CHEMOTHERAPY
1	7	Knee osteosarcoma	No
2	12	Acute myeloid leukemia	First cycle of G.A.T.L.A and a second cycle of ifosfamide and carboplatin etoposide, after relapse.
3	12	Hodgkin’s lymphoma	No
4	13	Perineal myosarcoma	Four cycles of ifosfamide, vincristine, actinomycin, epirubicin, etoposide and carboplatin.
5	13	Shoulder osteosarcoma	No
6	13	Inguinal synovial sarcoma	Three cycles of ifosfamide and doxorubicin.
7	14	Rib osteosarcoma	No
8	15	Hodgkin’s lymphoma	No
9	16	Hodgkin’s lymphoma	OPPA scheme, two cycles of vincristine, doxorubicin and procarbazine, COOP scheme and two cycles of vincristine, procarbazine and cyclophosphamide.
10	18	Ganglioneuroblastoma	Cycle 1, 2, 4 and 6 of cyclophosphamide, doxorubicin and vincristine; cycle 3 and 5 of etoposide and cisplatin; 6 cycles of 13-cys-retinoic. Also, she received 3000 cGy radiotherapy.
11	18	Hodgkin’s lymphoma	No
12	19	Myelodysplastic síndrome	Cytarabine, idarubicine and etoposide.

### Histology, measurement of follicular density and follicle classification

Before fixation in formalin, the fragment of tissue was measured in length, width and thickness for volume calculation. After 24 h fixation, fragments were embedded in paraffin, entirely cut into serial 5 μm sections and every fifth section was stained with hematoxylin-eosin (H&E) for routine histology. The remaining sections were kept for immunohistochemical staining. All ovarian follicles inside the whole tissue fragment were counted and classified according to Gougeon [17]. Only oocyte-containing follicles were included in the counting.

### Immunohistochemistry

Mounted paraffin sections were dewaxed in xylene, rehydrated in graded alcohols and washed in distilled water. Endogenous peroxidase activity was inhibited by using 0.5% H_2_O_2_/methanol (*v*/v) for 20 min at room temperature. Sections were then blocked for 1 h with 15% normal goat serum or normal rabbit serum in phosphate buffered saline (PBS) and incubated overnight at room temperature with the 1:200 diluted primary antibody: goat polyclonal anti-OCT3/4, goat polyclonal anti-VASA; goat polyclonal anti-BCL2; rabbit polyclonal anti-BAX; goat polyclonal anti-BID; rabbit polyclonal anti-MCL-1; rabbit polyclonal anti-FAS; rabbit polyclonal anti-FASL; rabbit polyclonal anti-PROCASPASE 3 and rabbit polyclonal anti-CLEAVED CASPASE 3. All antibodies were from Santa Cruz Biotechnology (Dallas, TX, USA) except cleaved CASPASE 3 purchased from AbCam (Cambridge, UK). After overnight incubation, slides were rinsed thrice in PBS and incubated for 1 h at room temperature with the appropriate 1:200-diluted biotinylated secondary antibody (Vector Labs, Peterborough, UK). After further washing in PBS, sections were incubated for 30 min with 1:100 diluted streptavidin-peroxidase complexes (ABC kit, Vector Labs, UK). Sections were then washed twice with PBS, and development of peroxidase activity was revealed with 0.05% 3,3′-diaminobenzidine (*w*/*v*) and 0.1% H_2_O_2_ (v/v) in Tris-HCl. Finally, sections were washed with distilled water and mounted in Canada balsam (Biopack, Buenos Aires, Argentina). Negative controls were processed simultaneously by omitting the primary antibody and/or preincubating the primary antibody with the specific commercial synthetic peptide.

### Western blot analysis of VASA, BCL2, BAX, MCL-1 and cleaved-BID proteins

Ovarian fragments preserved at − 80 °C were homogenized in ice-cold lysis buffer containing a protease inhibitor cocktail [0.5 mM phenylmethylsulfonyl fluoride (PMSF); 10 mM leupeptin; 10 mM pepstatin; 10 mM aprotinin], and centrifuged at 1.200 *g* at 4 °C for 10 min. The supernatant was collected and proteins were quantified using the Bradford Protein Assay (Bio-Rad Laboratories, Inc., Hercules, CA, USA). Total proteins (10 μg for BCL2, BAX and VASA; 20 μg for MCL-1 and cleaved-BID) from tissue extracts were separated by one-dimensional SDS-PAGE (10% for MCL-1 and VASA; 12% for BCL2, BAX and cleaved-BID) and then transferred onto polyvinylidene fluoride (PVDF) membranes (Immobilon-P Transfer membrane, Millipore, Billerica, MA, USA). Membranes were then blocked for 1 h in PBS + 0,1% Tween20 with bovine serum albumin (BSA) or non-fat dry milk depending on the first antibody to be used (3% BSA + 3% non fat dry milk for BAX; 5% non fat dry milk for BCL2, MCL-1 and cleaved-BID). After that, they were incubated 1 h at room temperature with the primary antibody (1400 diluted goat polyclonal anti-BCL2, 1:700 diluted rabbit polyclonal anti-BAX, 1:600 diluted rabbit monoclonal anti-cleaved-BID, 1:700 diluted rabbit polyclonal anti-MCL-1, all antibodies from Santa Cruz Biotechnology, Dallas, USA). Goat anti-rabbit IgG horseradish peroxidase-conjugated secondary antibody (Bio-Rad Laboratories, Inc., Hercules, CA, USA) was employed at a 1:3000 dilution or rabbit anti-goat IgG horseradish peroxidase-conjugated secondary antibody (Vector Labs, Peterborough, UK) at 1:5000 dilution. The immunoreactive product was visualized using the enhanced chemiluminescence system ECL plus GE (Amersham, Fairfield, Connecticut, USA). Snap-id (Millipore, Billerica, Massachusetts, USA) was employed to analyze VASA expression; a 1:200 dilution for goat polyclonal anti-VASA was used (Santa Cruz Biotecnology, Dallas, TX, USA). Horseradish peroxidase-conjugated second antibody was used at a 1:600 dilution. To confirm equal loading, each membrane was analyzed for β-actin protein expression with Snap-id, demonstrating that the band intensities did not show significant changes between the samples analyzed. Briefly, membranes were incubated with monoclonal anti-β-actin (Sigma, Saint Louis, Missouri, USA) diluted 1:20000. After washing, membranes were incubated with a goat anti-mouse IgG (Bio-Rad, 1:600) conjugated to peroxidase, and then revealed as described above. Stained protein molecular weight markers were used as standards (Fermentas, Vilnius, Lithuania). Densitometry was performed on Scion Image for Windows software (Scion Corporation 2000–2001) and VASA, BCL2, BAX, MCL-1 L, MCL-1S and BID expression was normalized to β-actin.

### RNA isolation and real time-PCR

Samples recovered in RNA later at the surgery room were maintained in that solution for 48 h and then stored a − 80 °C until used. Total ovary RNA was extracted with Trizol (Invitrogen, Waltham, MA, USA) according to the manufacturer’s instructions. Total RNA (3 μg) was treated with DNAse I (Invitrogen, Waltham, MA, USA) and used for reverse transcription in a 20 μl-reaction containing M-MLV reverse transcriptase (200 U/μl, Promega, Madison, WI, USA) and random hexamers primers (Biodynamics, Buenos Aires, Argentina). Reverse-transcribed cDNA was employed for quantitative polymerase chain reaction (PCR) using SYBR Green PCR Master Mix and specific forward (F) and reverse (R) primers (Table 3), in a Stratagene MPX500 cycler (Stratagene, La Jolla, CA, USA). Primers were used at a concentration of 0.3 μM in each reaction. The cycling conditions were as follows: step 1, 10 min at 95 °C; step 2, 30s at 95 °C; step 3, 30s at 55 °C; step 4, 30s at 60 °C, repeating steps 2 to 4 forty-five times. Data from the reaction were collected and analyzed by the complementary computer software (MxPro3005P v4.10 Build 389, Schema 85, Stratagene, La Jolla, CA, USA). Melting curves were run to confirm specificity of the signal. Relative quantitation of gene expression was performed using standard curves and normalized to β-actin in each sample. For assessment of quantitative differences in the cDNA target between samples, the mathematical model of Pfaffl was applied. The expression ratio was determined for each sample by calculating (E_target_)^ΔCt(target)^/(E_GβACTIN_)^ΔCt(βactin)^, where E is the efficiency of the primer set and CT is threshold cycle with ΔCt = Ct _(normalization cDNA)_ - Ct _(experimental cDNA)_. The amplification efficiency of each primer set was calculated from the slope of a standard amplification curve of log (ng cDNA) per reaction vs. Ct value (E = 10^-(1/slope)^). Efficiencies of 2 ± 0.1 were considered optimal.

**Table 3:** Oligonucleotide primers used for real-time PCR amplification of cDNA obtained after reverse transcription from adolescent human ovary RNA

GENE	PRIMERS (5′ → 3′) F: forward. R: reverse	AMPLIFIED PRODUCT (bp)
*BAX* (NM_138761.3)*	F: GCATCGGGGACGAACTGG R: GTCCCAAAGTAGGAGAGGA	307
*BCL2* (NM_000633.2)	F: GCCTTCTTTGAGTTCGG R: GGGTGATGCAAGCTCC	250
*BID* (NM_197966.2)	F: CCTTGCTCCGTGATGTCTTTC R: TCCGTTCAGTCCATCCCATTT	100
*MCL-1 L* (NM_021960.4)	F: TAAGGACAAAACGGGACTGG R: ACCAGCTCCTACTCCAGCAA	137
*MCL-1S* (NM_182763.2)	F: GAGACGGCCTTCCAAGGA R: ACCAGCTCCTACTCCAGCAA	112
*BCL-XL* (NM_138578.1)	F:GCAGGTATTGGTGAGTCGGATCGC R: CACAAAAGTATCCCAGCCGCCG	100
*FAS* (NM_000043.4)	F: CACTATTGCTGGAGTCAG R: CTGAGTCACTAGTAATGTCC	266
*FAS-L* (NM_000639.1)	F: TCAATGAAACTGGGCTGTACTTT R: AGAGTTCCTCATGTAGACCTTGT	101
*CASPASE-3* (NM_004346.3)	F: CCTCTTCCCCCATTCTCAT R: GAGTCCATTGATTCGCTTCC	119
*VASA* (NM_024415.2)	F: AGAAAGTAGTGATACTCAAGGACCAA R: TGACAGAGATTAGCTTCTTCAAAAGT	199
*OCT3/4 A* (NM_002701.3)	F: CTCCTGGAGGGCCAGGAATC R: CCACATCGGCCTGTGTATAT	341
*OCT3/4 B* (NM_203289.4)	F: ATGCATGAGTCAGTGAACAG R: CCACATCGGCCTGTGTATAT	263
*Β-actin* (NM_001101.3)	F: CTTCCCCTCCATCGTGGG R: GTGGTACGGCCAGAGGCG	357

### Terminal deoxynucleotidyl transferase-mediated dUTP nick-end labelling

Detection of DNA fragmentation was performed in formalin-fixed/paraffin-embedded sections by TUNEL technique, using the ‘In Situ Cell Death Detection Kit’ (Roche Diagnostics, Germany) with fluorescein-tagged nucleotides. The procedure followed the manufacturers’ recommendations. Treated sections were examined in an Olympus BX40 microscope by conventional epifluorescence with ultraviolet illumination. In order to confirm negative results, TUNEL-processed sections were incubated with 10 UI/ml DNase II (Sigma Chemical Co., USA) in 50 mM Tris–HCl, pH 7.5, 10 mM Mg_2_Cl and 1 mg/ml BSA for 10 min at room temperature. After incubation, slides were thoroughly rinsed and treated again according to the TUNEL protocol. Images were captured with an Olympus Camedia C-5060 camera.

### Statistical analysis

Mean and standard error (SEM) were calculated and the InfoStat Software (Version 2012, Grupo InfoStat, Universidad Nacional de Córdoba, Córdoba, Argentina) was used for one-way analysis of variance. A log_10_ transformation of data was done. Tukey’s test was used when differences between more than two groups were compared. A *p*-value of less than 0.05 was considered statistically significant.

## References

[1] Aitken RJ, Findlay JK, Hutt KJ, Kerr JB (2011). Apoptosis in the germ line. Reproduction..

[2] Albamonte MI, Albamonte MS, Stella I, Zuccardi L, Vitullo AD (2013). The infant and pubertal human ovary: Balbiani’s body associated VASA expression, immunohistochemical detection of apoptosis-related BCL2 and BAX proteins, and DNA fragmentation. Hum Reprod.

[3] Albamonte MS, Albamonte MI, Vitullo AD (2012). Germ line apoptosis in the mature human ovary. J Med Res Sci.

[4] Albamonte MS, Willis MA, Albamonte MI, Jensen F, Espinosa MB, Vitullo AD (2008). The developing human ovary: immunohistochemical analysis of the germ cell-specific VASA protein, BCL-2/BAX expression balance and apoptosis. Hum Reprod.

[5] Anderson R, Fulton N, Cowan G, Coutts S, Saunders P (2007). Conserved and divergent patterns of expression of DAZL, VASA and OCT4 in the germ cells of the human fetal ovary and testis. BMC Develop Biol.

[6] Anderson RA, McLaughlin M, Wallace WHB, Albertini DF, Telfer EE (2014). The immature human ovary shows loss of abnormal follicles and increasing follicle developmental competence through childhood and adolescence. Hum Reprod.

[7] Arends M, Wyllie A (1991). Apoptosis: mechanisms and roles in pathology. Int Rev Exp Pathol.

[8] Baker TG (1963). A quantitative and cytological study of germ cells in human ovaries. Proc Roy Soc B.

[9] Bhartiya D (2015). Ovarian stem cells are always accompanied by very small embryonic-like cells in adult mammalian ovary. J Ovarian Res.

[10] Bhartiya D, Patel H (2018). Ovarian stem cells, resolving controversies. J Assist Reprod Genet.

[11] Bhartiya D, Shaik A, Anand S, Patel H, Kapoor S, Sriraman K, Parte S, Unni S (2016). Endogenous, very small embryonic-like stem cells: critical review, therapeutic potential and a look ahead. Hum Reprod Update.

[12] Castrillon D, Quade B, Wang TY, Quigley C, Crum C (2000). The human vasa gene is specifically expressed in the germ cells lineage. Proc Natl Acad Sci.

[13] Cataldo NA, Dumesic DA, Goldsmith P, Jaffe BR (2000). Immunolocalization of FAS and FAS ligand in the ovaries of women with polycystic ovary syndrome: relationship to apoptosis. Hum Reprod.

[14] Chen W, Du J, Li X, Su J, Huang Y, Ding N, Zhang M, Jiang S (2017). miR-509-3p promotes cisplatin-induced apoptosis in ovarian cancer cells through the regulation of anti-apoptotic genes. Pharmacogenomics.

[15] Duncan FE, Pavone ME, Gunn AH, Badawy S, Gracia C, Ginsberg JP, Lockart B, Gosiengfiao Y, Woodruff TK (2015). Pediatric and teen ovarian tissue removed for cryopreservation contains follicles irrespective of age, disease diagnosis, treatment history, and specimen processing methods. J Adolesc Young Adult Oncol.

[16] Forabosco A, Sforza C, De Pol A, Vizzotto L, Marzona L, Ferrario F (1991). Morphometric study of human neonatal ovary. Anat Rec.

[17] Gougeon A (1996). Regulation of ovarian follicular development in primates: facts and hypotheses. Endocr Rev.

[18] Hartley PS, Bayne RAL, Robinson LLL, Fulton N, Anderson RA (2002). Developmental changes in expression of myeloid cell leukaemia-1 in human germ cells during oogenesis and early folliculogenesis. J Clin Endocrinol Metab.

[19] Hileman EO, Liu J, Albitar M, Keating MJ, Huang P (2004). Intrinsic oxidative stress in cancer cells: a biochemical basis for therapeutic selectivity. Cancer Chemother Pharmacol.

[20] Himelstein-Braw R, Peters H, Faber M (1977). Influence of irradiation and chemotherapy on the ovaries of children with abdominal tumours. Br J Cancer.

[21] Himelstein-Braw R, Peters H, Faber M (1978). Morphological study of the ovaries of leukaemic children. Br J Cancer.

[22] Johnson J, Canning J, Kanedo T, Pru JK, Tilly JL (2004). Germline stem cells and follicular renewal in the postnatal mammalian ovary. Nature.

[23] Kalich-Philosoph L, Roness H, Carmely A, Fishel-Bartal M, Ligumsky H, Paglin S, Wolf I, Kanety H, Sredni B, Meirow D (2013). Cyclophosphamide triggers follicle activation and “burnout”; AS101 prevents follicle loss and preserves fertility. Sci Transl Med.

[24] Kondo H, Maruo T, Peng X, Mochizuki M (1996). Immunological evidence for expression of the Fas antigen in the infant and adult human ovary during follicular regression and atresia. J Clin Endocrinol Metab.

[25] Lee J, Kim HK, Rho J, Han Y, Kim J (2006). The human OCT-4 isoforms differ in their ability to confer self-renewal. J Biol Chem.

[26] Leopardo NP, Jensen CF, Willis MA, Espinosa MB, Vitullo AD (2011). The developing ovary of the south American plains vizcacha, Lagostomus maximus (Mammalia, Rodentia): massive proliferation with no sign of apoptosis mediated germ cell attrition. Reproduction.

[27] Lintern-Moore S, Peters H, Moore GPM, Faber M (1974). Follicular development in the infant human ovary. J Reprod Fert.

[28] Peters H, Byskov AG, Grinsted J (1978). Follicular growth in fetal and prepubertal ovaries of humans and other primates. Clin Endocrinol Metab.

[29] Peters H, Himelstein-Braw R, Faber M (1976). The normal development of the ovary in childhood. Acta Endocrinol.

[30] Quirk SM, Cowan RG, Joshi SG, Kenrikson KP (1995). Fas-antigen mediated apoptosis in human granulosa/luteal cells. Biol Reprod.

[31] Schmidt KL, Byskov AG, Andersen AN, Andersen CY (2003). Density and distribution of primordial follicles in single pieces of cortex from 21 patients and in individual pieces of cortex from three entire human ovaries. Hum Reprod.

[32] Sforza C, Ferrario VF, de Pol A, Marzona L, Forni M, Forabosco A (1993). Morphometric study of the human ovary during compartmentalization. Anat Rec.

[33] Sharma A, Tripathi M, Satyam A, Kumar L (2009). Study of antioxidant levels in patients with multiple myeloma. Leuk Lymphoma.

[34] Stoop H, Honecker F, Cools M, de Krijger R, Bokemeyer C, Looijenga L (2005). Differentiation and development of human female germ cells during prenatal gonadogenesis: an immunohistochemical study. Hum Reprod.

[35] Vaskivuo T, Tapanainen JS (2002). Apoptosis in the human ovary. Reprod BioMed Online.

[36] Vaskivuo TE, Anttonen M, Herva R, Billig H, Dorland M, Velde ER, Stenbäck F, Heikinheimo M, Tapanainen JS (2001). Survival of human ovarian follicles from fetal to adult life: apoptosis, apoptosis-related proteins, and transcription factor GATA-4. J Clin Endocrinol Metab.

[37] Vetvicka V, Laganà AS, Salmeri FM, Triolo O, Palmara VI, Vitale SG, Sofo V, Králíčková M (2016). Regulation of apoptotic pathways during endometriosis: from the molecular basis to the future perspectives. Arch Gynecol Obstet.

